# Physicochemical studies of novel sugar fatty acid esters based on (*R*)-3-hydroxylated acids derived from bacterial polyhydroxyalkanoates and their potential environmental impact

**DOI:** 10.3389/fbioe.2023.1112053

**Published:** 2023-02-09

**Authors:** Wojciech Snoch, Ewelina Jarek, Dusan Milivojevic, Jasmina Nikodinovic-Runic, Maciej Guzik

**Affiliations:** ^1^ Jerzy Haber Institute of Catalysis, Surface Chemistry Polish Academy of Sciences, Kraków, Poland; ^2^ Institute of Molecular Genetics and Genetic Engineering, University of Belgrade, Belgrade, Serbia

**Keywords:** polyhydroxyalkanoates (PHA), sugar esters, cosmetic industry, *Caenorhabditis elegans*, environmental impact, surface activity

## Abstract

Sugar fatty acids esters are popular compounds widely used in both the nutritional, cosmetic and pharmaceutical industries due to their amphiphilic structure and consequent ability to reduce the surface tension of solutions. Furthermore, an important aspect in the implementation of any additives and formulations is their environmental impact. The properties of the esters depend on the type of sugar used and the hydrophobic component. In this work, selected physicochemical properties of new sugar esters based on lactose, glucose and galactose and hydroxy acids derived from bacterial polyhydroxyalkanoates are shown for the first time. Values for critical aggregation concentration, surface activity and pH make it possible that these esters could compete with other commercially used esters of similar chemical structure. The investigated compounds showed moderate emulsion stabilization abilities presented on the example of water-oil systems containing squalene and body oil. Their potential environmental impact appears to be low, as the esters are not toxic to *Caenorhabditis elegans* even at concentrations much higher than the critical aggregation concentration.

## Introduction

The demand for various emulsion systems and lubricants for skin care and protection is increasing every year (Farias et al., 2021). It became even more significant in recent years due to the fight against the COVID-19 pandemic–when the frequency of disinfection has become an inseparable part of everyone’s life (Masen, 2020; Patruno et al., 2020; Dini and Laneri, 2021). Therefore, the pharmaceutical and cosmetic industries are constantly working on perfect water-oil (W/O) systems that are less irritating to skin, are more stable in temperature and time, and are even capable of delivering bioactive compounds (Fabbron-Appas et al., 2021). At the same time, the impact of these systems on the environment is not without significance. Emulsions used in the industry should have physical and chemical parameters allowing them to be easily decomposed to not harm living organisms (Dini and Laneri, 2021; Tang et al., 2022). In order to meet these requirements a perfect combination of the oil phase and the emulsion stabilizer needs to be sought. Sugar fatty acid esters (SFAE) seem to be fair candidates for supporting such W/O systems. Their chemical structure and physicochemical properties, provide numerous applications in the pharmaceutical, cosmetic and food industries, including dietary supplements (Kumar, 2005; Łopaciuk and Łoboda, 2013; Blanc, 2015). The foremost important feature these chemicals offer in the formulation of products (i.e. creams, gels, foams, *etc.*) are their ability to decrease interfacial tension and to stabilize emulsions. Sometimes they are even accompanied by antimicrobial characteristics (Hill and Rhode, 1999; Van Kempen et al., 2013; Lucarini et al., 2016; Shao et al., 2018). These surfactant features can be manipulated either by the number of–OH groups within a sugar component, the length or modification of an aliphatic chain, which together can be described by hydrophilic-lipophilic balance (HLB) values (Sharma and Sarangdevot, 2012; Lémery et al., 2015; Pe et al., 2017). The hydrophobic component may be branched, include unsaturated bonds, additional hydroxylic groups or other desirable functionalities (van Kempen et al., 2014; Zhou et al., 2014; Riecan et al., 2022). Worth mentioning is a fact that global surfactant market size was 39,901 million USD in 2019 and is projected to grow to 52,417 million USD by 2025 (Tortilla et al., 2019).

Having the above in mind, our attention was drawn by a family of bacterial polyesters, namely polyhydroxyalkanoates (PHAs), as a source of easily modifiable hydroxyacids, used here as the hydrophobic component of SFAEs. Polyhydroxyalkanoates are synthesized by bacteria in response to environmental stress from various carbon sources and so far around 150 different building blocks are incorporated in their structure (Steinbiichel and Steinbiichel, 1995). The PHA monomers, namely (*R*)-3-hydroxylated fatty acids, are promising components for SFAEs synthesis with intrinsic antimicrobial and anticancer potential ⁠(Snoch et al., 2019; Snoch et al., 2021)⁠. Their structure–a hydroxyl group at the 3^rd^ position–allows for further modifications by decorating the molecule with the desired functionality (i.e. *via* an ether or an ester bond). Moreover, these sugar esters can be synthesized with an aid of biocatalysis, using enzymes such as lipases or esterases (Ansorge-Schumacher and Thum, 2013; Khan and Rathod, 2015; Pappalardo et al., 2017; Staroń et al., 2018), enabling preparation of true green additives. We have followed this path and employed biocatalysis in synthesis of our novel esters. However, little is known about their physicochemical characteristics.

**FIGURE 1:**
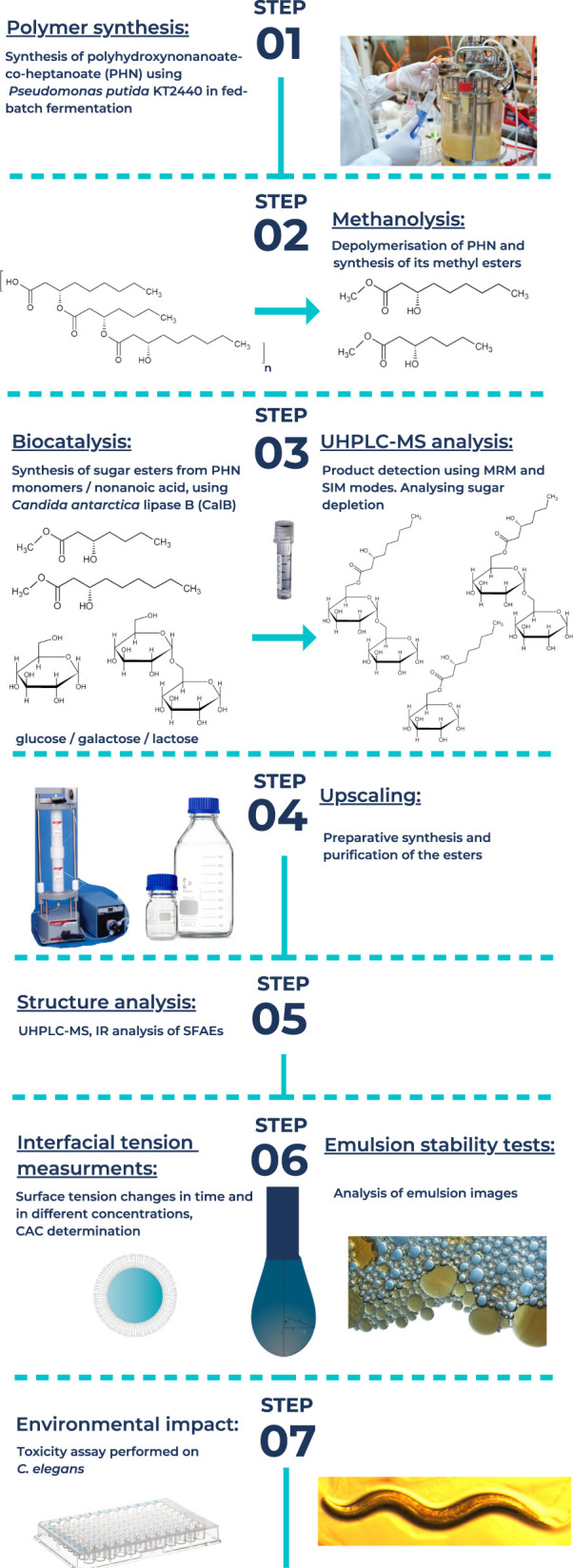
Scheme of the research and it's workflow.

This work describes surface activity of biotically synthesized sugar esters in comparison with their aliphatic counterparts (Figure 1). Their hydrophobic part was originated from bacterial poly–(*R*)–3–hydroxynonanoate–co–heptanoate (PHN). Firstly, we provide data on critical aggregation concentration (CAC), the point, conventionally chosen, where an increase of the surfactant concentration does not lead to a significant further reduction of the surface tension (Garofalakis et al., 2000) of the investigated compounds. Additionally, we performed simple tests of their emulsifying abilities with popular ingredients in cosmetic industry as skincare oil and squalene. Next, determination of emulsion stabilizing properties of these systems enabled us to verify industrial potential application of the SFAE as emulsifier. Finally, performing toxicity assay on *Caenorhabditis elegans* provided prognosis about the final key issue: potential environmental impact of the investigated stabilizers (Place, 2020; Ali and El-Ashry, 2021; Lanzerstorfer et al., 2021).

## Materials and methods

### Sugar esters used in this study

Sugar esters of aliphatic nonanoic acid along with a mixture of (*R*)-3-hydroxylated nonanoic acid and heptanoic acids (mPHN, derived from PHN) of glucose, galactose and lactose were obtained and characterized as described in our previous works. In search of effective anticancer agents—novel sugar esters based on polyhydroxyalkanoate Monomers (Snoch et al., 2021). The composition of new synthesized compounds is presented in Table 1, the yields and their purity are presented in Supplementary Table S1.

**TABLE 1:** Composition of enzymatically obtained sugar esters used in this work.

Compound name^a^	Type of mono/diester	Amount of mono/diester (%)	Mean molar mass (g mol^–1^)
C9-glu	c9	75.8	370.57
	c9c9	24.2	
C9-gal	c9	91.0	352.68
	c9c9	9.0	
C9-lac	c9	89.4	497.31
	c9c9	10.6	
mPHN-glu	c9	20.0	406.98
	c9c9	09.8	
	c7	16.3	
	c7c7	32.9	
	c9c7	21.0	
mPHN-gal	c9	20.5	401.61
	c9c9	27.0	
	c7	25.6	
	c7c7	15.0	
	c9c7	11.9	
mPHN-lac	c9	24.6	584.22
	c9c9	18.9	
	c7	06.3	
	c7c7	21.5	
	c9c7	28.8	

a) C9-glu: a mixture of mono and diesters of glucose nonanoate, C9-gal: a mixture of mono and diesters of galactose nonanoate, C9-lac: a mixture of mono and diesters of lactose non-aoate, mPHN-glu: a mixture of PHN monomers originated glucose mono and diesters, mPHN-gal: a mixture of PHN monomers originated from galactose mono and diesters, mPHN-lac: a mixture of PHN monomers originated lactose mono and diesters.
• c9-monoester containing one nine carbon atom chain • c7-monoester containing one seven carbon atom chain • c9c9-diester containing two nine carbon atom chains • c7c7-diester containing two seven carbon atom chains • c9c7-diester containing one nine and one seven carbon atom chain

### Determination of surface activity of synthesized compounds

The surface tension has been measured for different batches of synthetized compounds by using pendant drop shape analysis method by two apparatus. First apparatus: a home-made experimental set-up described in detail in (Para et al., 2006), with experimental error equals to 2 mN/m, was used. The Young–Laplace capillary equation was fitted to the digitally recorded drop image. Measured surface tension value corresponds to the value of the best fit (note: it is as the only unknown parameter in this equation). The dynamic surface tension measurements were performed every 5 s. Measured equilibrium surface tension corresponded to the obtained steady-state time after adsorption, which was depended on surfactant concentration. The studied solutions of surfactant were mixtures of sugars mono and diesters, which can differ by number of hydrophobic hydrocarbon chains. As a consequence, the kinetic curves for different drops of the same solution did not often overlap. That is why the experimental values for one solution are the mean values from all the recorder dynamic curves. As second apparatus a commercial tensiometer (PAT–1M, Sinterface, Berlin, Germany), with experimental error of 0.2 mN/m, was used. (Kairaliyeva et al., 2017). The PAT-1M apparatus allows for an accurate control of the droplet area (or its volume) by a syringe pump, driven by a feedback loop software based on the drop imaging. The dynamic interfacial tension *versus* time on a freshly formed drop is measured during the ageing of the interface while keeping the drop volume constant (11 µL). The equilibrium interfacial tensions are obtained from these data at long period of time. The greatest experimental error is mainly connected with small differences in composition from batch to batch so we present all experimental points and determined the critical aggregation concentration (CAC). All surface tension measurements were performed at 295 K. For all the experiments, ultrapure water—produced from the Millipore Direct-Q ® 5UV purification system (18 MΩ cm^−1^), was used. Its surface tension measurement provides a value of 72.5 ± 0.2 mN/m, stable for at least 2 hours at 20°C meaning negligible amount of surface-active impurities.

### Determination of hydrophilic- lipophilic balance

Hydrophilic-Lipophilic Balance was determined according Griffin method by using following equation (Griffin W.C., 1954)

$$HLB=20\;x\;\frac{hydrophilic\;group\;molecular\;weight}{total\;surfactant\;molecular\;weight}$$ (1)

### Emulsion stability

The ability of the SFAE to stabilize water–oil (W/O) systems was estimated by measuring time of phase separation in each performed emulsion. The experiment was conducted under following protocol: 5 g of each SFAE solutions were prepared as water phase and mixed with 0.5 g of oil phase: squalane or popular commercially available skincare oil- Bambino^®^ respectively. Concentrations of the SFAE in solutions were: 0.5 × CAC, 1.0 × CAC 1.5 × CAC respectively. The skincare oil consisted of: glycine soya oil, paraffinum liquidum, parfum, ethyl linolate, ethyl oleate, tocopherol, propylene glycol, propyl galate, citric acid, BHA, according to the manufacturer label in the unknown proportions. The mixtures were mixed in shaker at 25 C for 20 min and mixed vigorously with Vortex for another 1 min to ensure the phases were mixed sufficiently (An et al., 2019; Li and Xiang, 2019).

### Caenorgabditis elegans toxicity assay

Potential environmental impact of the investigated compounds was tested by treating *Caenorhabditis elegans* according to following protocol adapted from WormBook (Stiernagle, 2006) and (Djapovic et al., 2021). Briefly, synchronized worms (L4 stage) were suspended in a medium containing 95% M9 buffer (3.0 g of KH_2_PO_4_, 6.0 g of Na_2_HPO_4_, 5.0 g of NaCl, and 1 mL of 1 mol L^–1^ MgSO_4_ × 7 H_2_O in 1 L of water), 5% LB broth (10 g L^–1^ tryptone, 5 g L^–1^ yeast extract, and 10 g L^–1^NaCl), and 10 μg mL^–1^ of cholesterol. The experiment was carried out in 96-well flat-bottomed microtiter plates (Sarstedt, Nümbrecht, Germany) in the final volume of 100 μL per well. Suspension of nematodes (25 μL containing 25–35 nematodes) was transferred to the wells of a 96-well microtiter plate, where 50 μL of the medium was previously added. Next, 25 μL of a solvent control (DMSO) or 25 μL of a concentrated solution was added to the test wells. The examined esters were dissolved in DMSO to obtain stock solutions than added to the wells with worms. The final concentrations of the compounds were 2.0, 1.5, 1.0, 0.5, 0.25 and 0.125 mg mL^–1^. The final concentration of DMSO in each well was 1% (v/v) Subsequently, the plates were incubated at 25 C for 2 days. The fraction of dead worms was determined after 48 h by counting the number of dead worms and the total number of worms in each well, using a stereomicroscope (SMZ143-N2GG, Motic, Wetzlar, Germany). As a negative control experiment, nematodes were exposed to the medium containing 1% (v/v) DMSO.

## Results

After preparative synthesis, purification and drying the obtained SFAE were analyzed using UHPLC-MS (QQQ) in both selected ionic mass (SIM) and multiple reaction monitoring (MRM) modes. The obtained peaks from ESI+, i.e. (M + Na)^+^ adducts, were integrated so their peak areas enabled us to calculate fractions of mono and diesters. Mean molar masses of SFAE mixtures were calculated as well. The obtained results are presented below in Table 1.

**FIGURE 2:**
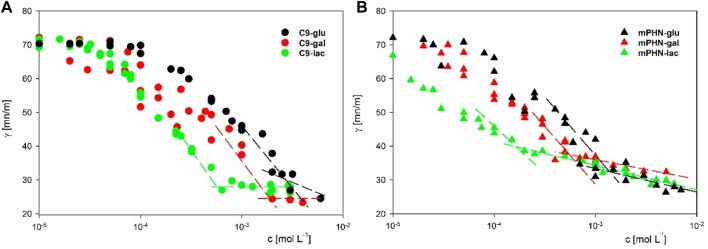
The surface tension dependency on surfactants concentration **(A)** C9-glu black circles; C9-gal red circles; C9-lac green circles **(B)** mPHN-glu black triangles; mPHN-gal red triangles; mPHN-lac green triangles. The intersection of the lines indicates the concentration at which the slope of the surface tension isotherms changes significantly, which is conventionally taken as CAC.

**FIGURE 3:**
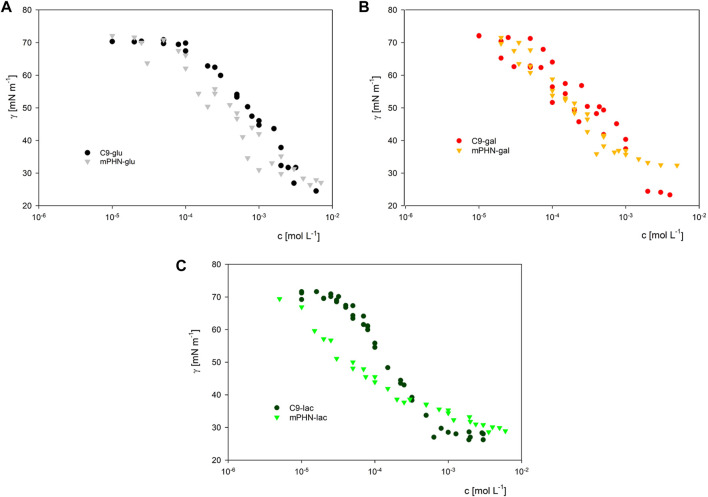
The comparison of surface activity of biocatalytically synthesized mPHN sugar esters with their aliphatic counterparts. Respectively for glucose (Panel A), galactose (Panel B) and lactose (Panel C) derivatives.

Instead of the critical micelle concentration (CMC), we decided to use the more general term—critical aggregation concentration (CAC)—because the LC-MS analysis shows that the synthesised compounds are in fact mixtures of mono and diesters with different percentage compositions (See Table 1) and undefined stereochemistry of the resulting esters. As the concentration of the surfactants tested increases, the surface tension does not transition sharply to a constant value, as is the case with pure mono-component surfactant solutions, but gradually changes marginally (Supplementary Figure S1). We used as CAC values the concentration at which the slopes of the curves connecting the experimental points significantly change shape and the surface tension for subsequent concentrations does not differ significantly (dashed lines in Figure 2). Despite the large scatter in the data obtained especially for the galactose derivatives, the experimental results of the solutions of the different batches, obtained on the two apparatus, coincide, and the trend in the differences in surface activity between the simple esters and mPHN derivatives is the same (Figure 2). The lactose derivatives are the most surface active, followed by galactose and the least glucose, as evidenced by the increasing values of the critical aggregation concentration (Table 2). A comparison of the aliphatic sugar esters and the corresponding mPHN derivatives shows that the mPHN derivatives are more surface active (Figure 3). In our case the quality of the anomers (α or β) in the tested solutions was not determined. Nevertheless, literature data indicate that the stereochemistry of the sugar derivatives can affect the surface tension. For example, *ß* anomers are more effective surfactants than α anomers, and differences in surface tension can be as high as 8 mN m^–1^. This is a result of differences in the ability to form intermolecular hydrogen bonds with other ester molecules and the surrounding water molecules (Nilsson et al., 1998). As a consequence, the sugar moieties’ hydrophilicity, surface activity and solubility are altered, which can result in the presence of relatively large aggregates in solution, but too fine (below 300 nm) to cause visible turbidity in solutions (Nilsson and So, 1998; Larsson et al., 2019). Clearly, this is an issue that will require additional research in the future.

**TABLE 2:** Physicochemical properties of the synthetized sugar esters and reference compounds.

Compounds used in this study								
Compound name	CAC	Surface tension γ	HLB griffin for each SFAE component					pH
	(mmol L–1)	(mN m–1)	c9	c7	c9c9	c7c7	c9c7	
C9-glu	2.7	31.5–24.5	11.25	–	7.83	–	–	5.39
C9-gal	2	25.2–23.5	11.25	–	7.83	–	–	5.33
C9-lac	0.56	29.2–25.8	14.2	–	11	–	–	6.29
mPHN-glu	1.4	35–26	11.72	12.79	8.7	9.81	9.22	3.92
mPHN-gal	0.56	37–32	11.72	12.79	8.7	9.81	9.22	4.12
mPHN-lac	0.016	39–28.5	14.41	15.27	11.49	12.57	12.01	6.8

Referenced compounds								
Compound name	CMC/CAC	Surface tension γ	HLB Griffin	References				
	(mmol L-1)	(mN m-1)						
octyl- B-D- glucoside	21.2	31	12.32	Gaudin et al. (2019)				
nonyl- B-D- glucoside	6.9	29.6	11.7					
Glucose monooctanoate (c8)	10.15	26.37	11.76	Zhang et al. (2015)				
Glucose monodecanoate (c10)	0.71	30.49	10.77					
Glucose monodecanoate (c10)	1.5	25.5	10.77	Hollenbach et al. (2020)				
Lactose caprate (c10)	2.5	40.6	14.8	Lee et al. (2018); Lucarini et al. (2018)				
Lactose laurate (c12)	0.55	40.4	14.1					
Sucrose Laurate (c12)	1.2	19.7	8.19	Ye et al. (2016)				
Sucrose oleate (c18)	0.0345	29.6	10.1					
Tween 80	0.01	38	15					
α-D-Glucose laurate (c12)	0.13	41.2	3.8	Garofalakis et al. (2000)				
α-D-Maltose laurate (c12)	0.12	35.9	4.5					
Lactose tetradecanoate (c14)	0.041	38.6	4.3					
Sucrose monooctanoate (c8) SM- 800 ^®^	6	28.7	15.8					
Sucrose monooctanoate (c10) SM- 1000 ^®^	0.57	32.9	–					
Sucrose monooctanoate (c12) SM- 1200 ^®^	0.29	33.5	–					

The pH of the solutions tested for simple sugar derivatives and mPHN-lac ranged from 5.2 to 6.8, which is characteristic of aqueous solutions in contact with carbon dioxide. Only in the case of mPHN-glu and mPHN-gal were the solutions slightly acidic (pH = 4.0 ± 0.2), so surface tension measurements were carried out for these solutions (C = 1 mmol L^–1^) in the presence of 0.1 mol L^–1^ NaCl. Increasing the ionic strength did not result in significant changes in surface tension (e.g. from 34.8 mN m^–1^ in H_2_0 to 33.1 mN m^–1^ in 0.1 mol L^–1^ NaCl for mPHN-gal and from 34.5 mN m^–1^ in H_2_0 to 32.6 in 0.1 mol L^–1^ NaCl), which are within the experimental limit of the apparatus error (2 mN m^–1^). This may indicate that surface-active ionic compounds are absent from the solution. In contrast, measurements in 0.1 mol L^–1^ NaOH solution resulted in a significant increase in surface tension (e.g. for mPHN-gal it increased to a value of 42 mN m^–1^ and for mPHN-gal to 58.6 mN m^–1^). This may be due to the different susceptibility to hydrolysis - the released organic acids dissociate in an alkaline environment and, as ionic surfactants, show much lower surface activity. In addition, the different degree of hydrolysis may be due to the different content of α and *ß* anomers.

Both glucose and galactose esters CAC values were much lower than the referenced CMC glucose monooctanoate (10.5 mmol L^–1^) and higher than glucose monodecanoate (0.71–1.5 mmol L^–1^) (Lee et al., 2018), which directly correlates with the chain length of the hydrophobic component of SFAE. Interestingly, C9-lac and mPHN-gal ester mixtures are somewhat identical to the commercial sucrose monodecanoate (SM-1000), the CMC of which was 0.56 mmol L^–1^. In general CACs of the mPHN derived esters obtained are higher than their counterparts in the literature, giving slightly lower interfacial tension values. The difference was observed for mPHN-lac which CAC is much lower than sucrose and lactose caprate (c10), laurate (c12) and even oleate (c18). Although mPHN-lac can be compare to Tween 80 (*γ* = 0.01 mmol L^–1^, 38 mN m^–1^). (Lee et al., 2018) (Lucarini et al., 2018) (Ye et al., 2016). All the synthesized based SFAE have their measured surface tension at the similar level (varying for the lowest of *γ* = 25.2–23.5 mN m^–1^ for C9-gal to the highest of *γ* = 32–28 mN m^–1^ for mPHN-lac, without any visible trend, Table 2). The HLB indexes calculated are lower for mono carbohydrates in both groups when compared to lactose esters. However, when hydrophobic component is considered, the HLB indexes are larger by 1–3 units for the aliphatic SFAEs. All the HLB values were in range of the referenced compounds (Table 2) regardless of whether the calculations took into account the content of mono- or diesters.

On the basis of the obtained interfacial tension profiles, it was possible to draw equilibrium concentrations curves, thus to determine aggregation concentrations (Figures 2A,B). The curves for different concentrations, but with a similar profile and little difference in values, are indicative of aggregation or micelle formation. They also provide indirect information on how the presence of mono- and diesters of c7 and c9 chains affects the ability to reduce surface tension. It is noticeable that the shape of each curve is depended on the sugar component (glucose and galactose vs. lactose esters, Figures 2A,B). In both cases the slopes of lactose esters were steeper than in these of glucose and galactose esters mixtures, which may be related to the difference in the size of the hydrophilic sugar heads. (Gaudin et al., 2019). Moreover, the slope of the curve for the mPHN-lac mixture was steeper than C9-lac (containing 89.4% monoester, 10.6% diesters), which may be related to the higher ratio of diesters in the mPHN-lac mixture (containing 30.8% of the total monoesters of c9 and c7 and 69.2% of the total of c9c9, c7c7, c9c7 diesters).

Taking into account the composition of the tested mixtures and contribution of diesters, we would expect lower ranges of CAC concentrations. Similar to those presented in Table 2 e.g. sucrose oleate. As can be seen, the presence of additional c9 or c7 chains in the hydrophobic component does not translate directly into properties comparable to twice as long aliphatic chains of other esters. It can be partially explained by the catalytic action of the lipase, which decorates a sugar moiety by attaching hydrophobic components on the opposite sides of the carbohydrate. There is also uncertainty when it comes to decorating sugars with (*R*)–3–hydroxylated fatty acids by the action of lipase, whether the final structure of the resultant SFAE is as described above for the aliphatic appendixes or it reassembles this of rhamnolipids (sugar + (*R*) –3-hydroxylated fatty acid + (*R*)–3-hydroxylated fatty acid). Moreover, the mere presence of hydroxyl group of monomers can also influence the branching of the entire molecule and disrupt the hydrophobicity of a carbon chain. (Hollenbach et al., 2020). Therefore, more detailed studies are needed in order to elucidated the final structure of the produced esters and also their behaviour on the molecular scale.

### Emulsion stability

Basing on a series of 5-min films and images taken up to 48 h of water/oil systems containing different concentrations of the tested SFAEs, it was not possible to measure the thickness of the emulsion layer and thus determine the stability index of the emulsion (Supplementary Figure S2). This was due to the short lifetime of the emulsion. However, depending on the type of ester, its concentration, and the composition of the oil phase, emulsion systems formed and maintained from several minutes to 1 hour (Table 3.). Emulsions containing squalene were less stable. W/O control systems containing no esters blurred after only a few minutes. On the other hand, systems containing SFAEs based on body care oil turned out to be more durable, as they had a diverse composition and contained co-surfactants such as alcohols. Furthermore, in most cases, differences in the durability and consistency of emulsions can be observed. They depend on the concentration of SFAEs in aqueous solutions. The higher the SFAE concentration, the more stable and homogeneous the system was. Systems containing C9-glu and C9-gal proved to be the most stable. The least stable system contained C9-lac. Emulsions based on mPHN-glu and mPHN-gal esters proved to be less stable than those originated from nonanoic acid. In contrast, the emulsions containing mPHN-lac exhibited a higher persistence than mPHN-glu, mPHN-gal and C9-lac. The maximum lifetime of the investigated homogeneous emulsion systems (Table 3 emulsion quality assessment 3, Supplementary Figure S2) was 60 min. These were systems containing a commercial baby care oil. However, some remaining emulsions were visible and even more stable: 180 min for mPHN-glu, mPHN-gal and mPHN-lac to 1440 min for C9-glu, C9-gal, C9-lac. In W/O systems that contained squalane, after 60 min, the quality rating were 2 or 1, respectively. These lifetimes are definitely too short to make them competitive against other surfactants used in the industry, such as Tween 80, Triton-X, Span 20, Span 60, polyethylene glycol (PEG). However, noteworthy is that commercially available products very often contain combinations of various surfactants e.g. Tween 80- polyoxoethylene sorbitan monooleate or Triton X-100-consisted of PEG and p-tert-octylphenol. Usually investigated W/O systems are based on combinations of two or more different ionic and/or nonionic surfactants and other co-surfactants i.e. alcohols and fatty acids, that make together the emulsions even more stable and extend their lifetime. (Watanabe et al., 2018). (Li and Friberg, 1982; McClements and Jafari, 2018). This approach should be investigated with our biocatalytically synthetized SFAE.

**TABLE 3:** Emulsion stability of two exemplary water/oil systems in time. Numbers-colors and their intensity is a scale referring to the intensity of a particular emulsion. Exemplary photos of the formed emulsions Supplementary Figure S2. a) Control- W/O systems with no SFAE addition b) squalene as oil phase with SFAEs as stabilizers c) baby care oil as an oil phase with SFAEs as stabilizers.

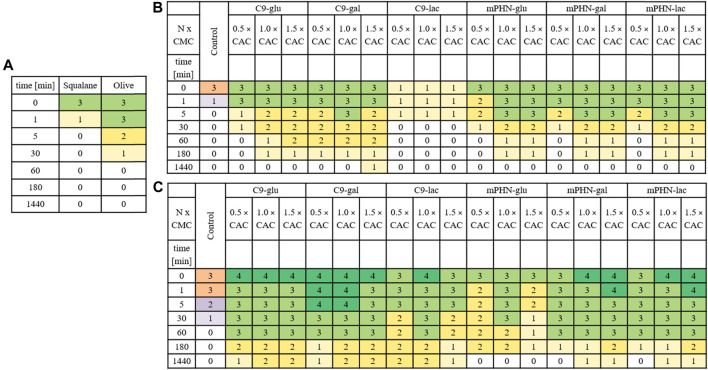

Although the expected lifetime of a commercially acceptable emulsion should be counted in days up to 1 month, the prepared here W/O systems did not perform well more than 24 h. We can explain this either by poor stabilizing properties of the SFAEs or their too-low concentrations used in preparation W/O systems, as well as lack of other co-surfactants. The calculated concentrations of the investigated surfactants present in the emulsions were between 0.001% and 1.0% m/v respectively (from 0.5 to 1.5 x CAC) while usually used concentrations of surfactants from the literature are between 0.1% and 5.0% m/v (Feng et al., 2022) but rarely calculated on their CAC or CMC values. That makes our system difficult to compare. Moreover, literature mentions several different techniques used for emulsion preparation i.e. shaking, ultrasound mixing, microwave pulsing, (Taha et al., 2020; Hyde et al., 2021), magnetic mixing, high pressure homogenization (Li and Xiang, 2019), and syringe mixing (Koursari et al., 2020). In recent years, even use of solid stabilizing particles (Pickering emulsions) became more popular. Nanomaterials derived from natural sources are an interesting alternative or supplementary for this application. (Velásquez-Cock et al., 2021). In order to compare our surfactants for their feasibility in applications other than described below (drug delivery purposes), studies should be conducted in greater concentrations and with other additives.

The ideal W/O system used in the cosmetic or pharmaceutical industry should arise from natural sources, and also should be able to form stable and durable emulsions. Emulsions need to be stable enough to be stored at room temperature. Most importantly, they should be biodegradable and biocompatible. When it comes to drug delivery systems, their life time longer than 48–72 h is not advisable either. Another crucial factor is emulsion bioaccessibility. That means easy absorption by the epithelial surfaces, passing through cell membranes but not damaging them. (Production of green surfactants: Market prospects | Elsevier Enhanced Reader) The micelles protecting the structure of the drug from enzymes and/or pH changes should be able to release it gradually to the tissues. (Felzenszwalb et al., 2019). The micelle-building components ought to be easily degraded or removed from the body and easily decomposed in the environment. (Tovar-Sanchez et al., 2020) (Hunt, 2017) (Fagan and Portman, 2014). Undoubtedly, the studied SFAEs show some emulsion-stabilizing properties, but in order to give these W/O systems the desired longer lifespan, further optimization of SFAEs concentration, oil phase composition, mixing methods and addition of co-surfactants is required. (McCartney et al., 2019). The emulsion stabilizing properties of the investigated SFAE may not be spectacular, nevertheless sometimes desired if there is a need of administration of a less stable formulation to a patient prepared minutes prior injection/topical application. The used SFAE were already shown to exhibit anticancer properties, which increase their possibility to be applied in the medical industry (Tuvia et al., 2014).

### Environmental impact of sugar fatty acid esters

**FIGURE 4:**
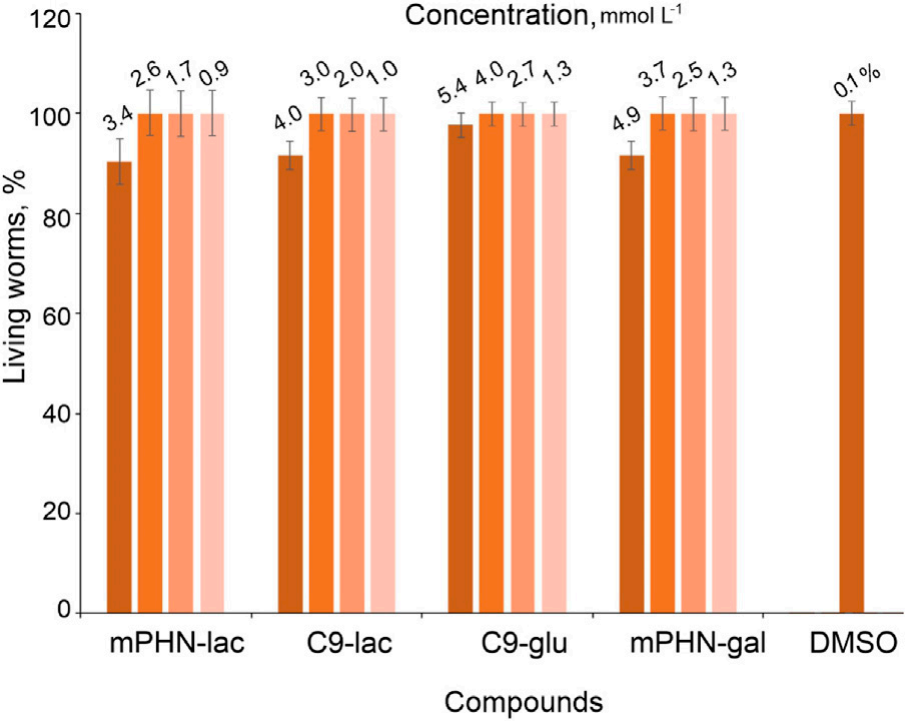
Toxicity of the sugar esters against *Caenorhabditis elegans* after 48 h exposure.

*Caenorhabditis elegans* is a multicellular, non-parasitic model organism that is a valuable research object for testing the effects of various industrial chemicals such as anti-cancer drugs and antibiotics (Judy et al., 2019; Wittkowski et al., 2019). Every year, mankind supplies a huge amount of industrial wastewater and with it–surfactants (Production of green surfactants: Market prospects | Elsevier Enhanced Reader; Akbari et al., 2018; Felzenszwalb et al., 2019; Tovar-Sanchez et al., 2020). Therefore, there is a need for continuous monitoring of their impact on organisms living in soil and groundwaters (Ebele et al., 2017; Rathi et al., 2021). The usefulness of nematodes representatives is manifested in a fast life cycle, easy multiplication and obtaining a large number of individuals in subsequent generations, the possibility of long–term storage of larvae and eggs in laboratory conditions. They do not require continuous breeding, and the procedure for synchronizing the life cycles of individuals in a population is simple. In addition, *Caenorhabditis elegans* feed on an easily available source of food–bacteria *E. coli*. The most important from the human point of view, is the presence of simple organ systems: nervous (nerve ring), blood, protonfridial, gonads, and the ability to assess not only the size of the population under the microscope, but also the ability to actively move individuals in the population. Therefore, nematodes can be indirect bioindicators of the influence of the tested substances on the natural environment (Fagan and Portman, 2014; Hunt, 2017). Having the above in mind, toxicity of the tested sugar esters against *Caenorhabditis elegans* was assessed by observing the nematodes under a microscope after 48 h of exposure. Based on the mobility of *Caenorhabditis elegans* their viability was assessed. Actively moving organisms were considered living, non-moving organisms were considered dead, and barely moving organisms were considered alive as well (having in mind, that the compounds could have a negative effect on worms) (Figure 4).

Observations of the nematodes allowed us to conclude that the obtained SFAEs do not pose a major threat to *Caenorhabditis elegans* at the given time of exposure. Only the highest concentrations (2.0 mg ml^–1^) of the mixtures reduced nematodes populations up to 15% but 1.5 mg ml^–1^ was already not effective. The concentrations of the esters per the well ranged from 0.0625 to 2.0 mg ml^–1^ which correspond to their molar concentrations of: C9-glu 0.186.14–5.69 mmol L^–1^; C9-gal 0.162–5.312 mmol L^–1^; C9-lac 0.125–4.0 mmol L^–1^; mPHN-glu 0.142–4.544 mmol L^–1^; mPHN-gal 0.148–4.744 mmol L^–1^; mPHN-lac 0.119–3.816 mmol L^–1^, respectively. We have previously reported that SFAE esters based on nonanoic acid and PHN monomers show anticancer potential. The reported consternations (IC_50_) of these compounds that were found to be effective against certain cancer lines (In Search of Effective Anticancer Agents—Novel Sugar Esters Based on Polyhydroxyalkanoate Monomers-W. Snoch et al., 2021. pdf) are at the levels of being non-toxic to *Caenorhabditis elegans*. For example, the effective IC_50_ for Du145 and HTB140 cell lines after 24 h exposures to all tested SFAE were below 0.25 mg ml^–1^ (i.e. IC_50_ range respectively 0.09–1.5 mmol L^–1^for cancer cells and 0.5–2.5 mmol L^–1^, respectively for reference healthy cells).

Sugar esters belong to the group of non-ionic surfactants. The presence of a hydrophobic fatty acid tail and a hydrophilic sugar head give them an amphiphilic character. The chemical structure itself does not, in general, pose a direct threat to the entire organism of nematode. However, this amphiphilicity and the ability of compounds to lower the surface tension of solutions may permeabilize cell membranes (Tuvia et al., 2014; McCartney et al., 2019). On the other hand, the presence of long carbon chains (such as c18 steric acid) increases the solubility of individual compounds in water (e.g. in industrial wastewaters) (Vinarov et al., 2018). All this together causes the entry of undesirable substances from the environment into the cells, an increase in the concentration of H^+^ ions, free oxygen radicals, herbicides, pesticides or salts (which can cause an osmotic shock), which in turn may direct the cells to the apoptotic pathway (Shao et al., 2018; Cavanagh et al., 2019; Appah et al., 2020; Liu et al., 2021; Martins-Gomes et al., 2022). In addition, the hydrophobic components of the SFAE tested do not exceed 9 carbon atoms, which may be another advantage compared to other commonly used esters based on acids with a chain length of c12, c16 or c18 (e.g. Span 20, Span 80, PEG 20– Sorbitan monolaurate (SPEG), Tween 20, Tween 80). Since increasing the chain length of the hydrophobic component also increases the toxicity of SFAE (Bunchongprasert and Shao, 2020). For comparison, the toxicity of other structurally similar allose-based esters to *Caenorhabditis elegans* with various carbon chain lengths n = 2, 4, 6, 8 was in the between 0.2 and 1.0 mmol L^–1^ (Sakoguchi et al., 2019).

In addition, every cosmetic or drug carrier potentially used in the industry must be thoroughly tested in terms of toxicology, before it goes to preclinical research (the smallest, largest harmful dose, and chronic administration of the compound), at every possible stage, not only cellular, but also more complex living organisms (Dent et al., 2018; Bopp et al., 2019). The highest tested levels of the SFAE (2.0 mg ml^–1^ around 6.0 mmol L^–1^) that reduced *Caenorhabditis elegans* populations ∼15% were near to CMC values. At the same time, the range of surfactants concentrations used in the water phase, to design model emulsion systems, were 0.5–10-fold CAC. Information obtained about minor negative effects of the esters on tested nematode in this concentration range opens the possibility for further application trials. However, it should be remembered that the SFAE molecules building micelles have a different concentration and organization than those dispersed in the buffers in which the *Caenorhabditis elegans* were used. They may behave differently and show a different level of toxicity, therefore more detailed investigation is foreseen for the future (Khamkar, 2011; Kaur et al., 2016; Rusanov, 2018; Bunchongprasert and Shao, 2020).

## Conclusion

The physicochemical properties of the obtained sugar esters were characterized which enabled their application potential evaluation. The obtained CAC and surface tension values correspond to compounds with a similar structure from the literature (such as SM-800, lactose caprate, or glucose monodecanoate). Also, the ability to create water–oil systems based on popular cosmetic ingredients, such as squalene and the commercial skincare oil confirmed application potential of the tested esters. However, the process of forming and testing these emulsions should be further optimized. It would be necessary to test a series of different oil components, as well as co-surfactants, in order to compare their emulsion stability indexes, size of the micelles formed and their behaviour.

In future prospective, it is worthy to focus on assessing the toxicity of not only the compounds themselves, but entire SFAEs based stable emulsion systems. Extending these experiments to such aspects as influence the of SFAE on increasing the vulnerability of *Caenorhabditis elegans* for these emulsion systems and repeat it in unfavourable water and soil conditions (presence of pesticides, inorganic salts, low pH, osmotically active substances). Even assessing susceptibility to opportunistic organisms would be valuable. From pharmaceutical and medical points of view, a key step is to answer questions about the mechanisms of the esters interaction with cells and/or model organisms, including the permeabilization of cells and intracellular membranes, and the ability to reversibly modulate endothelial electrical resistance (TEER), would be of great value. (Lucarini et al., 2018) Nematodes should be examined in more detail in terms of the impact of SFAE, and emulsion systems stabilized by them, on their internal organs, such as the endocrine and nervous systems, and also the ability to reproduce. (Ebele et al., 2017; Alfhili et al., 2018)

## Data Availability

The original contributions presented in the study are included in the article/Supplementary Material, further inquiries can be directed to the corresponding author.
