# Supplementary material for: Physicochemical studies of novel sugar fatty acid esters based on (R)-3-hydroxylated acids derived from bacterial polyhydroxyalkanoates and their potential environmental impact
Source: Front Bioeng Biotechnol. 2023 Feb 9;11:1112053. doi: 10.3389/fbioe.2023.1112053 (PMC9947713; doi:10.3389/fbioe.2023.1112053)
Supplement: Supplementary file 1 [file Presentation1.pptx]

## Slide 1
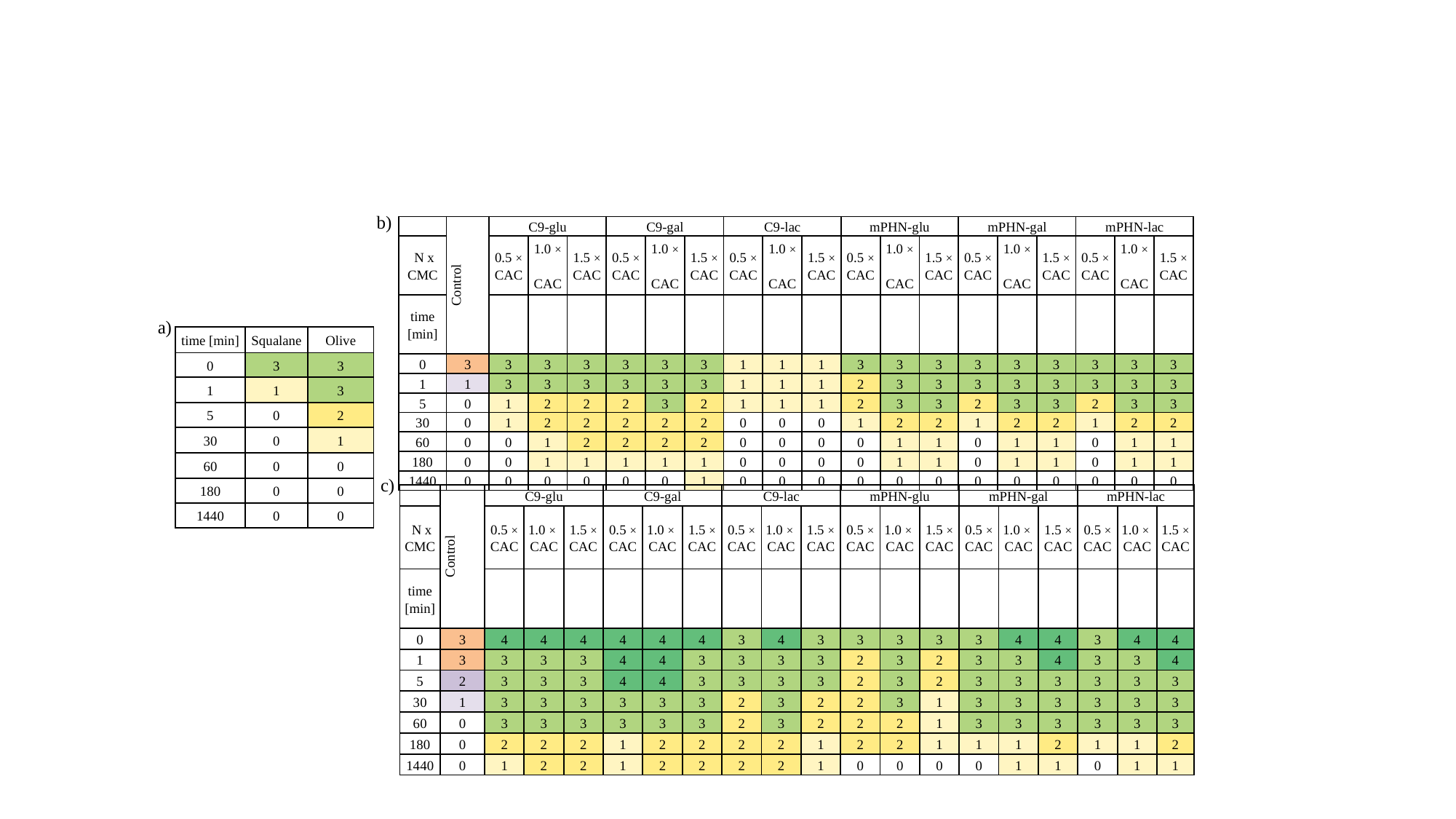

b)
| | Control | C9-glu | | | C9-gal | | | C9-lac | | | mPHN-glu | | | mPHN-gal | | | mPHN-lac | | |
| --- | --- | --- | --- | --- | --- | --- | --- | --- | --- | --- | --- | --- | --- | --- | --- | --- | --- | --- | --- |
| N x CMC | | 0.5 × CAC | 1.0 × CAC | 1.5 × CAC | 0.5 × CAC | 1.0 × CAC | 1.5 × CAC | 0.5 × CAC | 1.0 × CAC | 1.5 × CAC | 0.5 × CAC | 1.0 × CAC | 1.5 × CAC | 0.5 × CAC | 1.0 × CAC | 1.5 × CAC | 0.5 × CAC | 1.0 × CAC | 1.5 × CAC |
| time [min] | | | | | | | | | | | | | | | | | | | |
| 0 | 3 | 3 | 3 | 3 | 3 | 3 | 3 | 1 | 1 | 1 | 3 | 3 | 3 | 3 | 3 | 3 | 3 | 3 | 3 |
| 1 | 1 | 3 | 3 | 3 | 3 | 3 | 3 | 1 | 1 | 1 | 2 | 3 | 3 | 3 | 3 | 3 | 3 | 3 | 3 |
| 5 | 0 | 1 | 2 | 2 | 2 | 3 | 2 | 1 | 1 | 1 | 2 | 3 | 3 | 2 | 3 | 3 | 2 | 3 | 3 |
| 30 | 0 | 1 | 2 | 2 | 2 | 2 | 2 | 0 | 0 | 0 | 1 | 2 | 2 | 1 | 2 | 2 | 1 | 2 | 2 |
| 60 | 0 | 0 | 1 | 2 | 2 | 2 | 2 | 0 | 0 | 0 | 0 | 1 | 1 | 0 | 1 | 1 | 0 | 1 | 1 |
| 180 | 0 | 0 | 1 | 1 | 1 | 1 | 1 | 0 | 0 | 0 | 0 | 1 | 1 | 0 | 1 | 1 | 0 | 1 | 1 |
| 1440 | 0 | 0 | 0 | 0 | 0 | 0 | 1 | 0 | 0 | 0 | 0 | 0 | 0 | 0 | 0 | 0 | 0 | 0 | 0 |
a)
| time [min] | Squalane | Olive |
| --- | --- | --- |
| 0 | 3 | 3 |
| 1 | 1 | 3 |
| 5 | 0 | 2 |
| 30 | 0 | 1 |
| 60 | 0 | 0 |
| 180 | 0 | 0 |
| 1440 | 0 | 0 |
c)
| | Control | C9-glu | | | C9-gal | | | C9-lac | | | mPHN-glu | | | mPHN-gal | | | mPHN-lac | | |
| --- | --- | --- | --- | --- | --- | --- | --- | --- | --- | --- | --- | --- | --- | --- | --- | --- | --- | --- | --- |
| N x CMC | | 0.5 × CAC | 1.0 × CAC | 1.5 × CAC | 0.5 × CAC | 1.0 × CAC | 1.5 × CAC | 0.5 × CAC | 1.0 × CAC | 1.5 × CAC | 0.5 × CAC | 1.0 × CAC | 1.5 × CAC | 0.5 × CAC | 1.0 × CAC | 1.5 × CAC | 0.5 × CAC | 1.0 × CAC | 1.5 × CAC |
| time [min] | | | | | | | | | | | | | | | | | | | |
| 0 | 3 | 4 | 4 | 4 | 4 | 4 | 4 | 3 | 4 | 3 | 3 | 3 | 3 | 3 | 4 | 4 | 3 | 4 | 4 |
| 1 | 3 | 3 | 3 | 3 | 4 | 4 | 3 | 3 | 3 | 3 | 2 | 3 | 2 | 3 | 3 | 4 | 3 | 3 | 4 |
| 5 | 2 | 3 | 3 | 3 | 4 | 4 | 3 | 3 | 3 | 3 | 2 | 3 | 2 | 3 | 3 | 3 | 3 | 3 | 3 |
| 30 | 1 | 3 | 3 | 3 | 3 | 3 | 3 | 2 | 3 | 2 | 2 | 3 | 1 | 3 | 3 | 3 | 3 | 3 | 3 |
| 60 | 0 | 3 | 3 | 3 | 3 | 3 | 3 | 2 | 3 | 2 | 2 | 2 | 1 | 3 | 3 | 3 | 3 | 3 | 3 |
| 180 | 0 | 2 | 2 | 2 | 1 | 2 | 2 | 2 | 2 | 1 | 2 | 2 | 1 | 1 | 1 | 2 | 1 | 1 | 2 |
| 1440 | 0 | 1 | 2 | 2 | 1 | 2 | 2 | 2 | 2 | 1 | 0 | 0 | 0 | 0 | 1 | 1 | 0 | 1 | 1 |
