# Supplementary material for: Physicochemical studies of novel sugar fatty acid esters based on (R)-3-hydroxylated acids derived from bacterial polyhydroxyalkanoates and their potential environmental impact
Source: Front Bioeng Biotechnol. 2023 Feb 9;11:1112053. doi: 10.3389/fbioe.2023.1112053 (PMC9947713; doi:10.3389/fbioe.2023.1112053)
Supplement: Supplementary file 2 [file DataSheet1.docx]

**“Physicochemical studies of novel sugar fatty acid esters based on (*R*)-3-hydroxylated acids derived from bacterial polyhydroxyalkanoates and their potential environmental impact”**

Wojciech Snoch ^1^, Ewelina Jarek^1^, Dusan Milivojevic^2^, Jasmina Nikodinovic-Runic^2^, Maciej Guzik^1*^

^1^ Jerzy Haber Institute of Catalysis and Surface Chemistry, Polish Academy of Sciences, Niezapominajek 8, 30-239 Kraków, Poland

^2^ Institute of Molecular Genetics and Genetic Engineering (IMGGE) University of Belgrade

*** Correspondence:**Maciej Guzik
[maciej.guzik@ikifp.edu.pl](mailto:maciej.guzik@ikifp.edu.pl); Tel.: +48-12-6395-159

Keywords: polyhydroxyalkanoates, sugar esters, cosmetic industry, Nematoda, environmental impact

**SUPPLEMENTARY**

**Table S1. Purity, efficiency and yields of the obtained SFAE.**

| **Compound name:** | **Mas of the obtained ester [g]** | **Purity [%]** | **Mass expected if 100% was monoester [g]** | **Yield 1 [%]** | **Mass expected if 100% was diester** | **Yield 2 [%]** |
| --- | --- | --- | --- | --- | --- | --- |
|  |  |  |  |  |  |  |
| **C9-glu** | 0.328 | 84.78 | 2.779 | 10.01 | 3.995 | 6.96 |
| **C9-gal** | 0.278 | 84.45 | 2.779 | 8.44 | 3.995 | 5.87 |
| **C9-lac** | 0.135 | 94.59 | 2.092 | 6.10 | 2.700 | 4.73 |
| **mPHN-glu** | 0.593 | 87.73 | 1.25 | 41.53 | 3.124 | 16.65 |
| **mPHN-gal** | 0.6 | 89.05 | 1.25 | 42.67 | 3.124 | 17.10 |
| **mPHN-lac** | 0.118 | 93.86 | 1.87 | 5.93 | 2.436 | 4.55 |


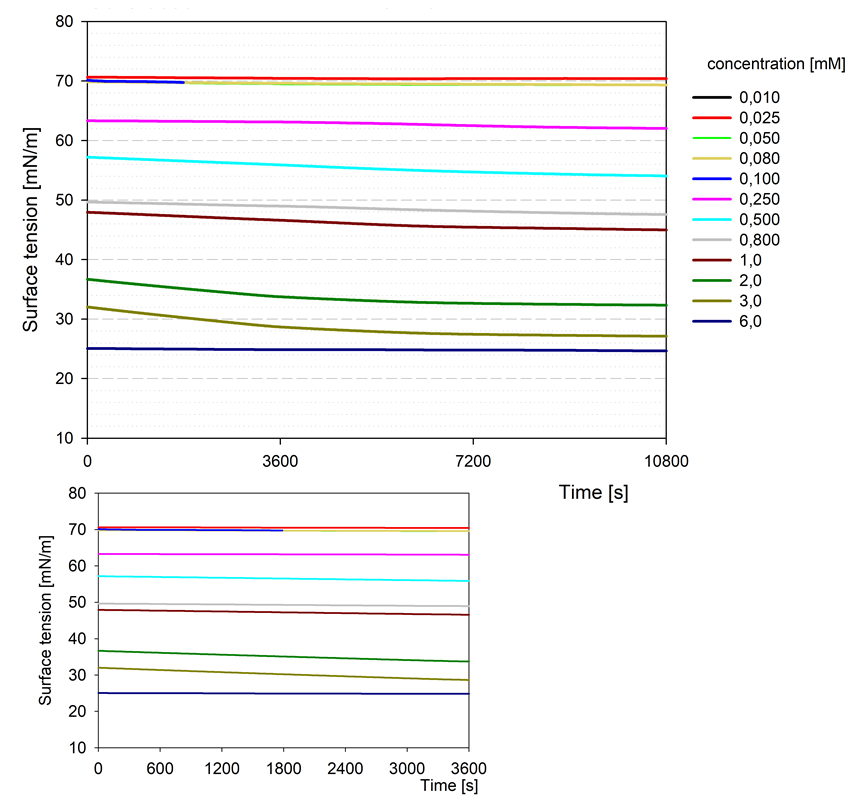

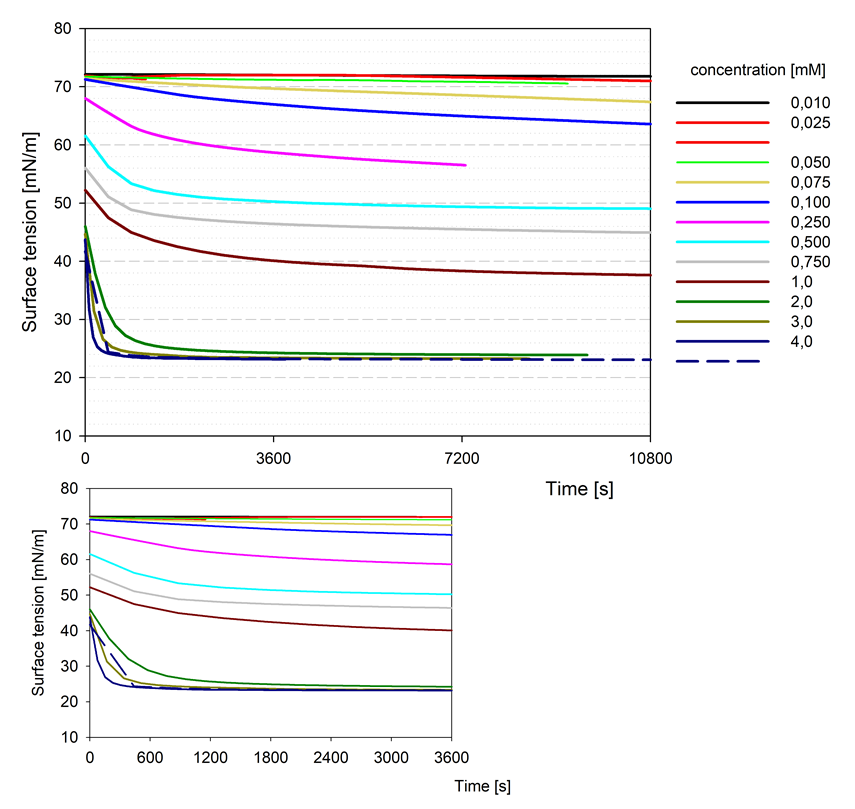


1. C9-glu b) C9-gal


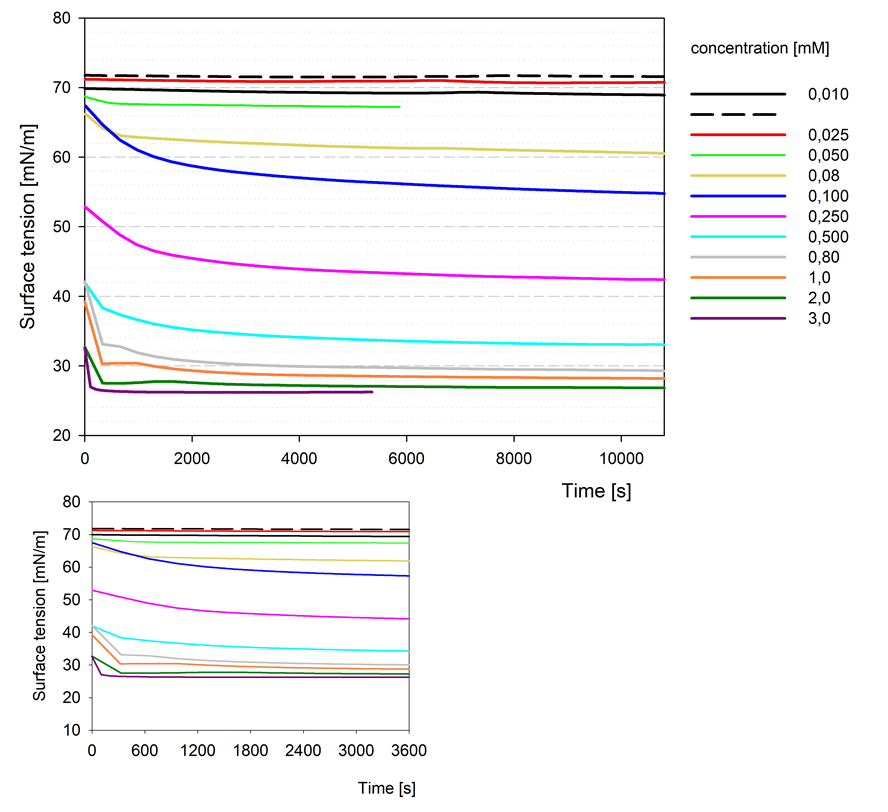

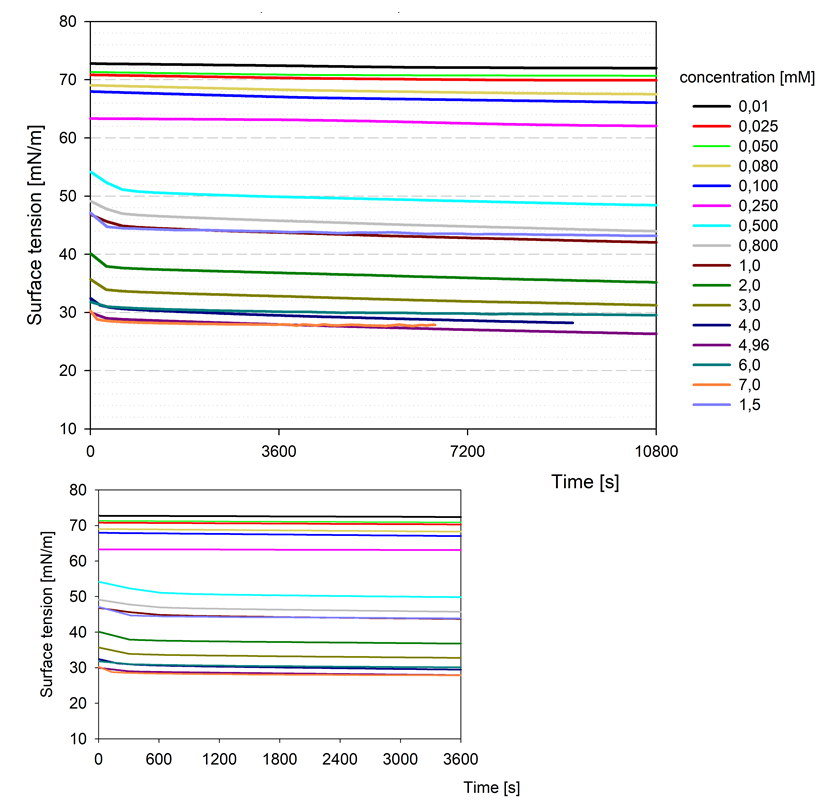


c) C9-lac d) mPHN-glu


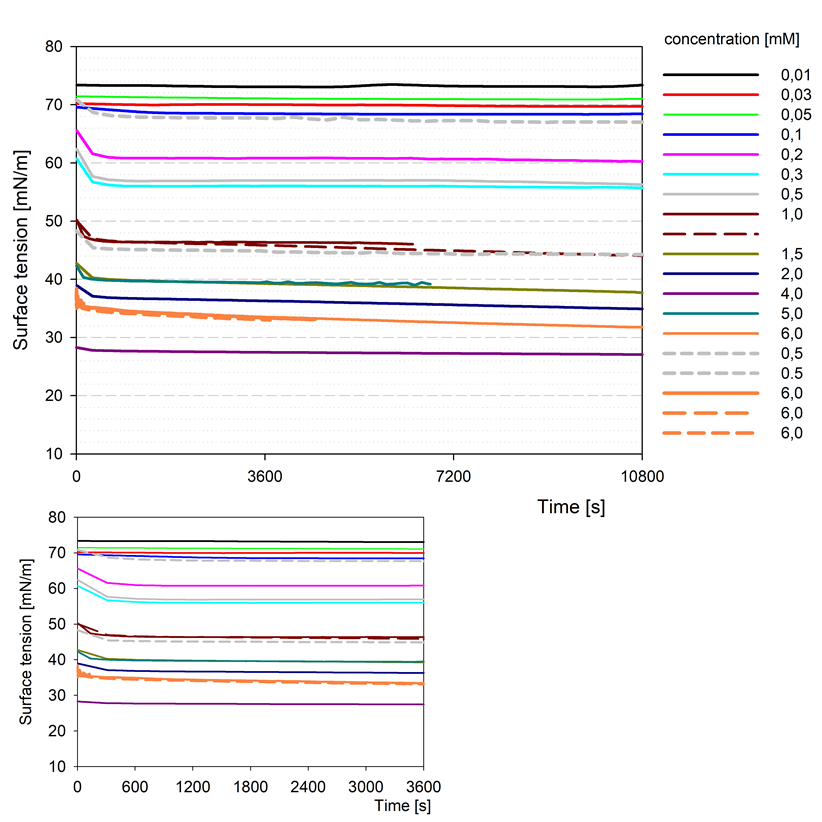

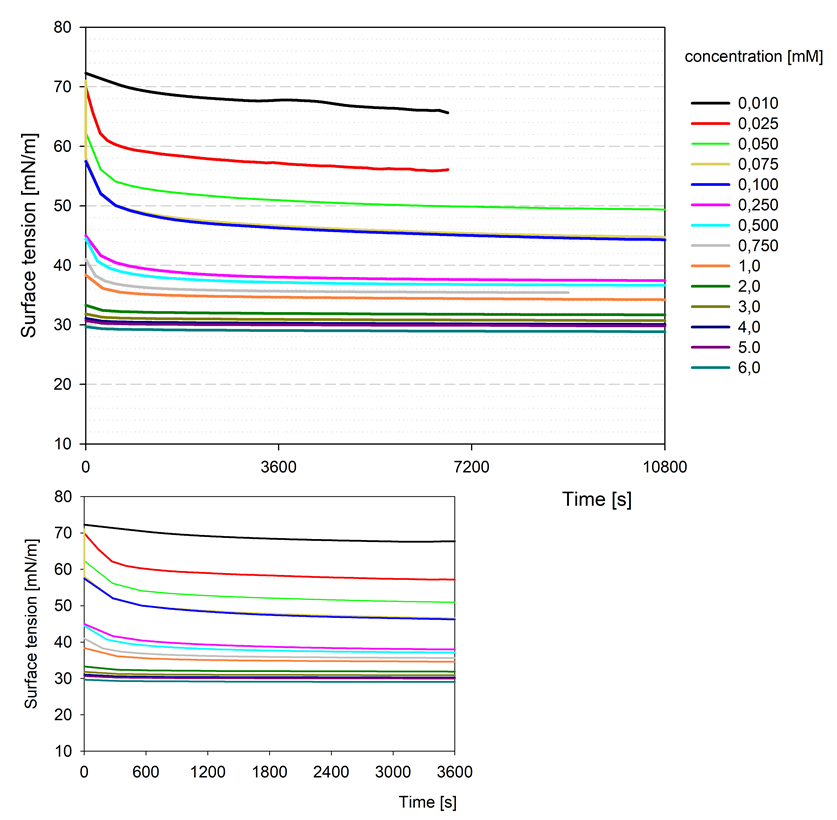


e) mPHN-gal f) mPHN-lac

**Fig S1. Interfacial tension changes in time depending on sugar ester concentration**

| 0 | 1 | 2 | 3 | 4 |
| --- | --- | --- | --- | --- |
| 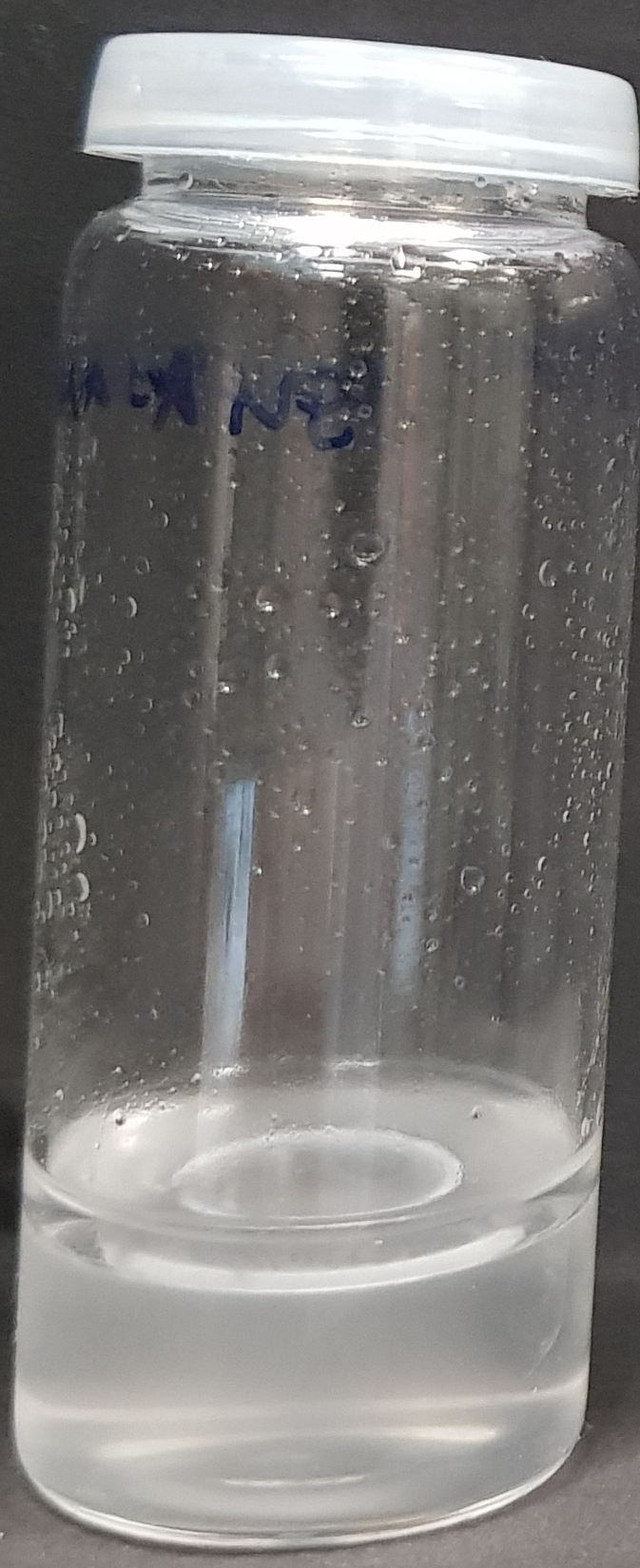 | 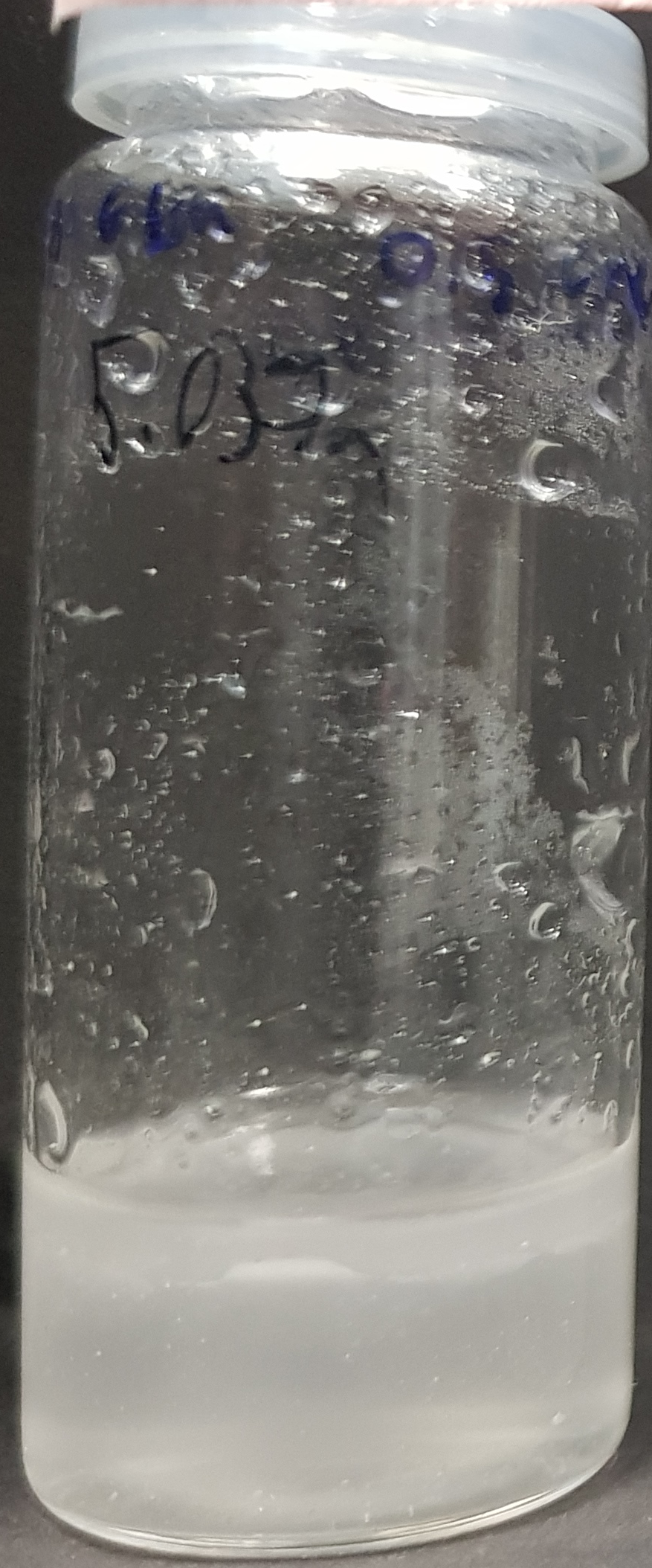 | 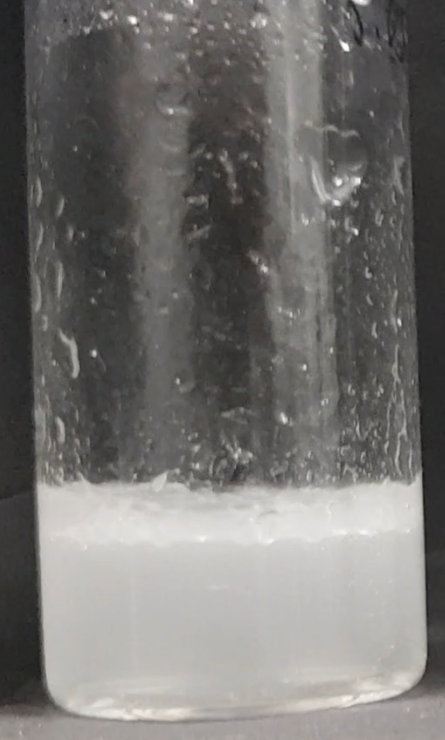 | 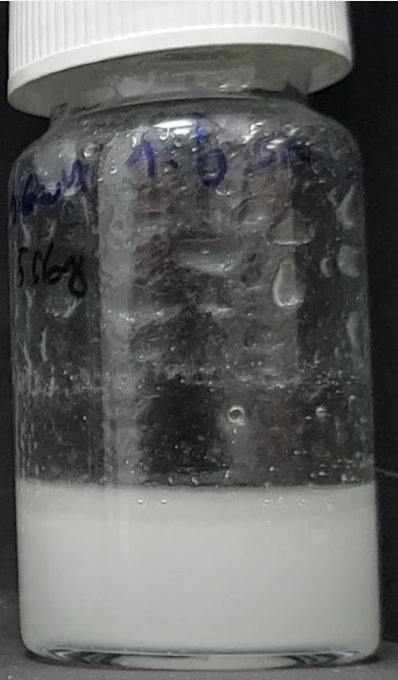 | 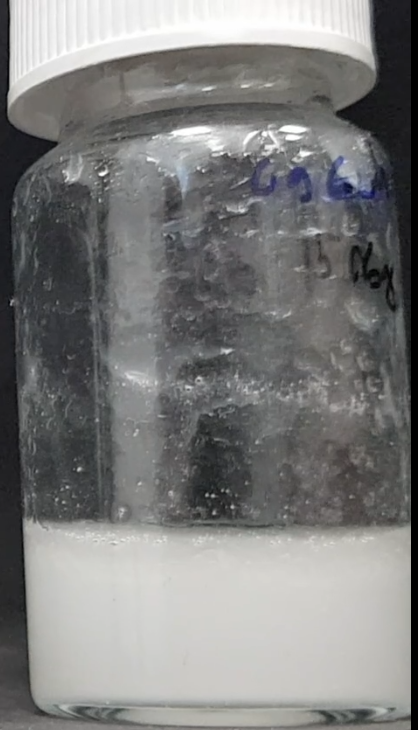 |
| C9-glu squalane- before start making emulsion | C9-glu squalene, 30 min, 0.5 × CAC | C9-glu squalene, 5min, 0.5 × CAC | C9-glu olive 1min, 1.5 × CAC | C9-glu olive 0min, just after mixing, 1.5 × CAC |
| 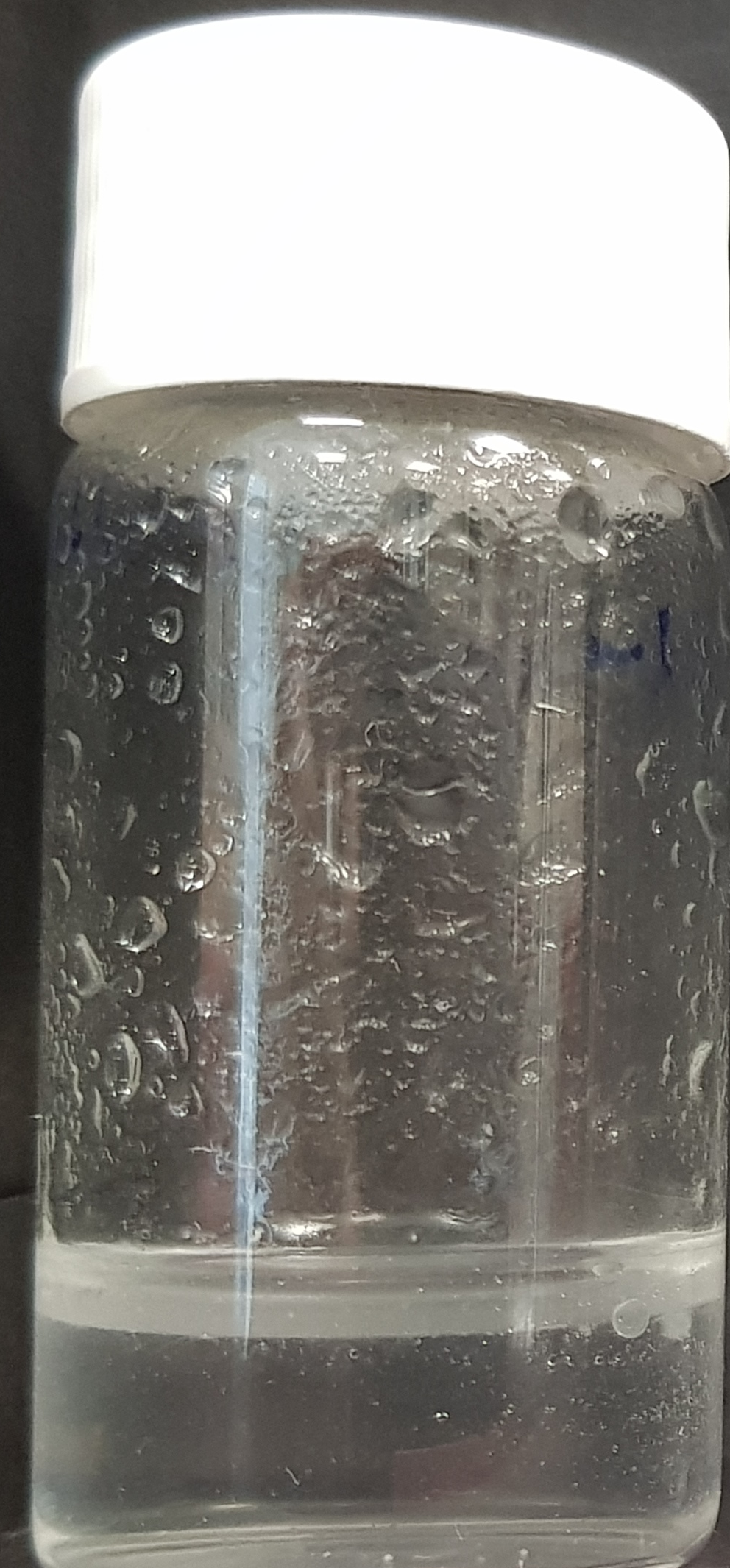 | 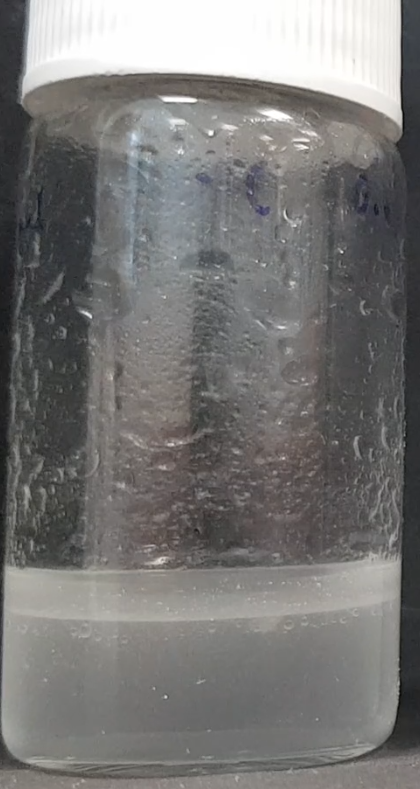 | 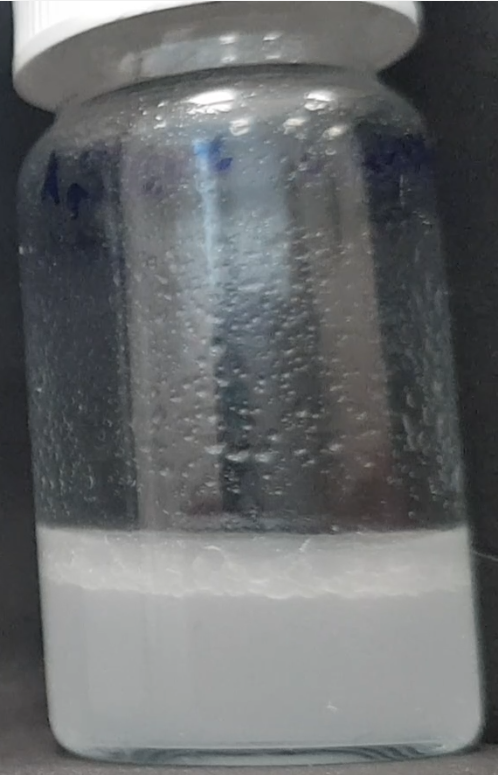 | 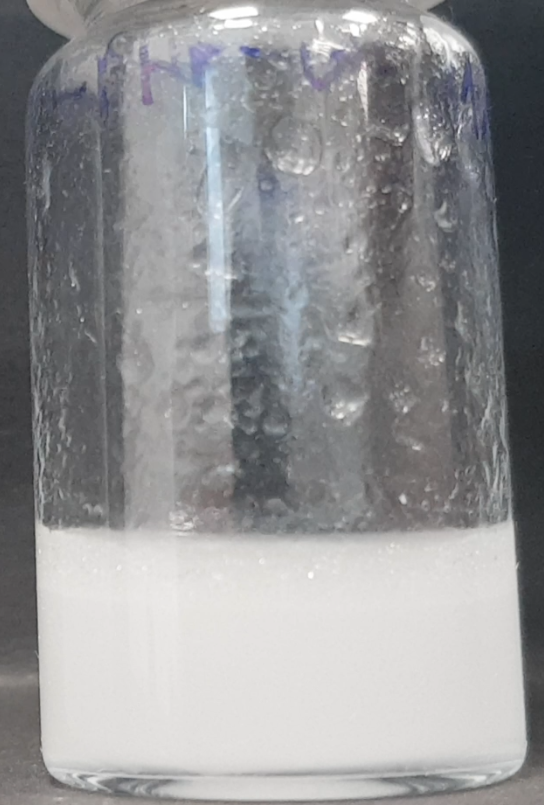 | 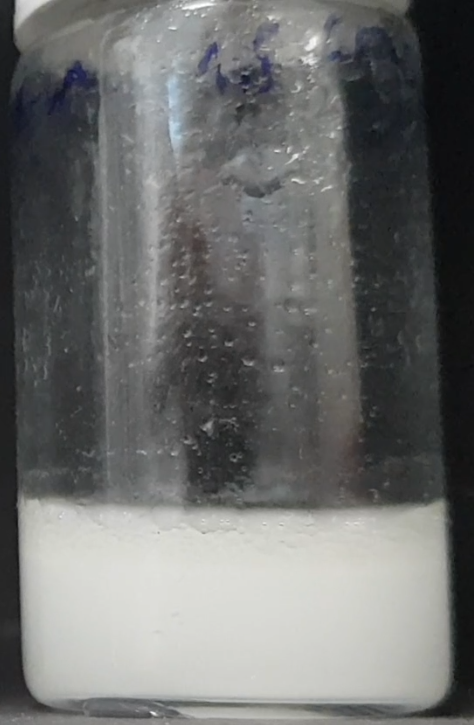 |
| mPHN-lac squalane- after 24h | mPHN-lac squlane 30 min, 1.0 × CAC | mPHN-lac squalene 5 min 1.5 × CAC | mPHN-lac squalane 0 min | mPHN-lac olive 0 min |

**Figure S2. Exemplary photos of the formed emulsions stabilized by SFAE**
